# Genome-wide linkage analysis of systolic blood pressure slope using the Genetic Analysis Workshop 13 data sets

**DOI:** 10.1186/1471-2156-4-S1-S86

**Published:** 2003-12-31

**Authors:** Dushanthi Pinnaduwage, Joseph Beyene, Shafagh Fallah

**Affiliations:** 1 Division of Epidemiology and Biostatistics, Samuel Lunenfeld Research Institute, Mount Sinai Hospital, Toronto, Ontario, M5G 1X5, Canada; 2 Litwin Centre for Cancer Genetics, Mount Sinai Hospital, Toronto, Ontario, M5G 1X5, Canada; 3 Program in Population Health Sciences, Research Institute, Hospital for Sick Children, Toronto, Ontario, M5G 1X8, Canada; 4 Department of Public Health Sciences, University of Toronto, Toronto, Ontario, Canada

**Keywords:** longitudinal data, rate of change, multiple imputation, linkage

## Abstract

Systolic blood pressure (SBP) is an age-dependent complex trait for which both environmental and genetic factors may play a role in explaining variability among individuals. We performed a genome-wide scan of the rate of change in SBP over time on the Framingham Heart Study data and one randomly selected replicate of the simulated data from the Genetic Analysis Workshop 13. We used a variance-component model to carry out linkage analysis and a Markov chain Monte Carlo-based multiple imputation approach to recover missing information. Furthermore, we adopted two selection strategies along with the multiple imputation to deal with subjects taking antihypertensive treatment. The simulated data were used to compare these two strategies, to explore the effectiveness of the multiple imputation in recovering varying degrees of missing information, and its impact on linkage analysis results. For the Framingham data, the marker with the highest LOD score for SBP slope was found on chromosome 7. Interestingly, we found that SBP slopes were not heritable in males but were for females; the marker with the highest LOD score was found on chromosome 18. Using the simulated data, we found that handling treated subjects using the multiple imputation improved the linkage results. We conclude that multiple imputation is a promising approach in recovering missing information in longitudinal genetic studies and hence in improving subsequent linkage analyses.

## Background

The Framingham Heart Study (FHS), with its extensive longitudinal data structure, has been a key source of information over the last several decades, allowing researchers to understand and elucidate associations between risk factors and cardiovascular diseases [1]. One of the physiological measurements recorded over the course of the study is systolic blood pressure (SBP), a complex phenotype that may be influenced by both environmental and genetic factors. A number of studies have found multiple regions on the genome that may contain candidate genes responsible for variability in SBP. A genome-wide scan of SBP in the Quebec Family Study [2] found evidence for linkage to regions on chromosomes 1, 2, 5, 7, 8, and 19. Using highly discordant full sibling design, Krushkal and colleagues [3] reported significant linkage to regions on chromosomes 2, 5, 6, and 15. A recent genome-wide scan of blood pressure in the Mexican Americans found suggestive linkage for SBP to regions on chromosomes 18 and 21 [4]. Levy et al. [5] reported linkage to the ACE region on chromosome 17 using the Framingham Heart Study data. They performed a genome-wide linkage analysis for SBP using 332 large families from this study. The longitudinal phenotype used in their analysis was the residual from a model that regressed within-subject mean SBP on the subject's mean age and mean body mass index. In this paper, we consider an alternative approach in which we use the rate of change in systolic blood pressure over time (SBP slope) as the phenotype of interest in a genome-wide linkage analysis using a variance-component model and a multiple imputation (MI) approach as the preferred method of replacing missing longitudinal measurements. MI was further used to deal with subjects taking antihypertensive treatment.

## Methods

Cohort 2 of the FHS data and one randomly selected replicate (Replicate 19) of the Genetic Analysis Workshop 13 simulated data were used. We used both complete and missing data of Replicate 19. All data sets contain up to five repeated measurements per individual except for the complete data of Replicate 19, which contain all measurements.

One of the challenges in calculating the slope phenotype was the presence of missing age and SBP measurements. Conventional methods for dealing with missing data include *complete-case analysis *and *single imputation*. In complete-case analysis, any variable with at least one missing value will lead to exclusion of a subject from the analysis no matter how complete other variables might be. This approach may result in considerable bias and loss of statistical efficiency. The single imputation methods are also problematic. By replacing a missing data point with a single imputed value, such as the average, one ignores the uncertainty inherent in the predictions of missing values. MI [6] remedies the problems associated with the conventional approaches. This is a computer-intensive method in which missing data are replaced with a set of plausible values where random variation is deliberately introduced in the imputation process. This random imputation process is repeated a number of times producing multiple "completed" data sets.

A Markov chain Monte-Carlo based MI [7], which assumes multivariate normality of the data, was used to impute missing values in SBP and age. Following recommendations in the missing data literature, we imputed five sets of data. For each "completed" data set, we obtained slopes from simple linear regression of SBP on age for each individual and these slopes were then averaged across the five imputations, and used for linkage analysis.

Apart from handling missing values in general, we used MI to handle subjects treated for hypertension. We adopted two strategies: 1) Method 1: imputing missing values, after excluding subjects who died and those who received antihypertensive treatment at any of the visits, and 2) Method 2: imputing, after excluding subjects who died but including those who were treated. In Method 2, imputation was done by censoring SBP measurements after the first antihypertensive medication and then imputing subsequent values based upon pre-treatment rate of change.

Although we do not have information on specific cause of death, we suspected that the likelihood of having extreme SBP values might be higher among the deceased. Since the MI method we adopted in this paper relies on the assumption of multivariate normality, which is a non-robust distribution to extreme cases, we decided to exclude deceased subjects from both methods. We outline a possible alternative approach to deal with these subjects in the discussion section.

The FHS data was used in an attempt to identify genes that contribute to the variation of the longitudinal SBP as measured by the slope. We took advantage of the simulated data, using both *complete *and *missing *data of Replicate 19, in order to assess the effectiveness of MI on linkage analyses results. Furthermore, we compared the performances of Method 1 and Method 2.

The SBP slopes obtained from Method 1 and Method 2 were used as the phenotype for genome-wide linkage analyses. Both two-point and multipoint variance-component linkage analyses were performed including gender, body mass index, grams of alcohol per day, and number of cigarettes smoked per day as covariates. Since each of these covariates was measured over time, the average measurements were used at the individual level. Heritability estimates for the slopes were also obtained.

After removing treated and dead individuals, we deliberately introduced missingness to the complete data from Replicate 19 to investigate the effectiveness of MI in recovering missing data for longitudinal linkage studies. We investigated four scenarios: 1) 10% of subjects missing only one measurement, 2) 10% of subjects missing two or more measurements, 3) 25% of subjects missing only one measurement, and 4) 25% of subjects missing two or more measurements. The linkage results obtained from the complete data of Replicate 19 were used as the reference values to compare with results obtained from the above four scenarios.

MI was performed using the SAS statistical software, version 8.02 (SAS Institute Inc., Cary, North Carolina). SOLAR (Southwest Foundation for Biomedical Research, San Antonio, Texas) was used for calculating heritability estimates and for the genome-wide linkage analysis using a variance-component method [8]. All analyses of the simulated data were conducted without knowledge of the generating model.

## Results

Inspection of individual profile plots relating SBP with age suggests that summarizing the longitudinal data using a linear slope would be reasonable. Thus, all results reported here pertain to analyses of slopes from simple linear regression models of SBP on age, calculated for every individual separately. The distribution of the calculated slopes appears symmetric for both the Framingham and Replicate 19 of the simulated data sets.

There were 1672 subjects in Cohort 2 of Framingham data. For Cohort 2 of Replicate 19 there were 1632 subjects for both complete and missing data. For the two-point IBD calculations, we used 330 pedigrees with 4692 subjects (i.e., including Cohort 1, the original cohort) for both data sets. The final number of subjects remained for the analyses from Method 1 (removing treated and deceased) and Method 2 (including treated but excluding deceased) for Framingham and simulated data are given in Table 1 and Table 2, respectively.

The distributional summary statistics for SBP slopes and heritability values are shown in Table 1 and Table 2. The mean slopes for males and females were significantly different for the Framingham data (*p*-value = 0.0003) but not for the simulated data. Furthermore, heritability increased from 0.23 (males and females combined data) to 0.37 for females. As a result, we performed sex-specific linkage analyses in addition to the overall linkage for the Framingham data. For the sex-specific analysis we used slopes generated from Method 2.

Results of two-point genome scan analyses for overall and sex-specific Framingham data are summarized in Table 3. In the overall analysis, we obtained a maximum LOD score of 2.24 at marker GATA24D12 on chromosome 7. In the sex-specific analysis, no LOD score > 1.0 was detected for males. In contrast, a maximum LOD score of 2.25 was found on chromosome 18 at marker ATA82B02 for females.

**Figure 1:**
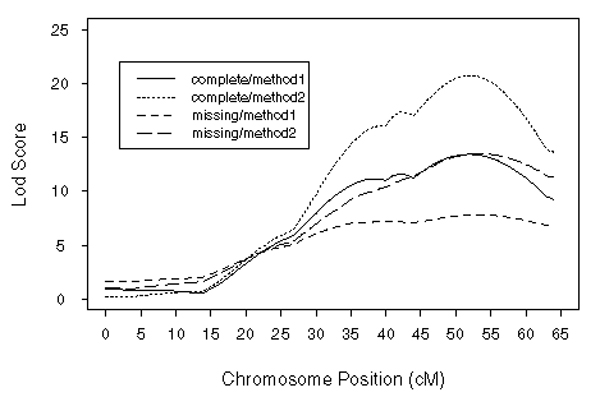
Multipoint LOD score profiles for chromosome 21 in the complete and missing simulated data sets, using Method 1 and Method 2

For Replicate 19 of the simulated data (complete and missing), we found very high two-point maximum LOD scores on chromosome 21 by both Method 1 and Method 2. The LOD scores on all of the other chromosomes were < 2.0. The two-point LOD scores are not shown in this paper but Figure 1 shows multipoint LOD score profiles for both complete and missing data by both methods. As can be seen from this figure, the complete data with Method 2 resulted in the highest maximum LOD score (20.8), whereas the lowest maximum LOD score (7.8) was obtained from the missing data with Method 1. These two scenarios illustrate the best-case (complete data, Method 2) and worst-case (missing data, Method 1) scenarios, respectively. A quantitative trait loci (QTL) was detected at position 52 cM with complete data and at 54 cM for missing data. In fact, the position of one of the major slope genes (s10) in the simulated data is at position 53.6 cM according to the generating model. It appears that MI combined with Method 2 provides increased power to detect linkage compared with Method 1.

Linkage results with and without MI in the presence of varying degree of missing information are summarized in Table 4. The results show that the linkage estimates yielded using MI were close to those obtained from the complete data (reference values). When the percentage of missing data is low, the results of complete-case analysis (without MI) yielded estimates similar to the reference values but as the amount of missing information increased, the maximum LOD score changed and its location deviated from the reference values. Further, the efficiency of MI also decreased as the amount of missing information increased, but not to the extent of the case without MI.

## Discussion

We conducted this study to detect QTLs affecting variability of SBP slopes. Suggestive evidence for linkage was found on chromosome 7 at marker GATA24D12 for the Framingham data when we used the combined sample of males and females. In the sex-specific analyses, a different QTL was detected on chromosome 18 at marker ATA82B02 in females but not in males. This could be due to several reasons such as sex differences in the slopes, different sex-specific penetrances at loci, or epistatic interaction between loci on autosomes and sex chromosomes. It is also possible that the male SBP slopes are determined environmentally not genetically since their slopes are not heritable (*p*-value = 0.17).

For the simulated data, we found one of the slope genes on chromosome 21 but failed to detect any other slope genes. Both Method 1 and Method 2 were able to find linkage within 2 cM of the correct location of the true slope gene with the largest effect. The multipoint LOD scores obtained by Method 1 were considerably lower than those obtained from Method 2 for both complete and missing data. Our Method 2 approach was able to improve on linkage results from Method 1 because it was able to incorporate clinically and genetically important information into subsequent linkage analyses by including treated people. With Method 1, sample size was reduced because of the exclusion of treated subjects, and hence power to detect linkage was decreased. Although we were able to find the gene with the largest effect size in the simulated data using Method 1, we may fail to detect a gene with moderate effect size.

Both Method 1 and Method 2 have the disadvantage of losing information from deceased individuals. These people may be the most informative for genetic analysis since they could have died from diseases for which SBP is a risk factor. With the limited number of observations in Cohort 2, and the reliance on multivariate normality assumption by the multiple imputation method, we concluded that including these possibly extreme cases might invalidate our approach and therefore decided to exclude them from analysis. However, it would be interesting to consider robust alternatives such as the heavy-tailed multivariate t distribution that may be more appropriate to incorporate outlying cases. As far as we know, such methods are not yet available in the MI literature.

In the examination of the effect of the number of missing values on MI, and hence on the linkage results, we noticed that MI recovered a reasonable amount of information from missing data. Missing observations are prevalent in large longitudinal genetic studies such as the Framingham Heart Study. We believe that MI (multiple imputation) is a promising approach in recovering missing information in a large data set with a moderate amount of missing information.

## Figures and Tables

**Table 1:** Distribution and heritability of SBP slope for Framingham data

	**Overall**		**Sex-Specific (Method 2)**	
		
	**Method 1**	**Method 2**	**Male**	**Female**
*N*	1276	1579	770	809
Range	(-2.35, 4.12)	(-2.35, 4.12)	(-2.35, 4.12)	(-2.15, 3.15)
Std. Dev.	0.76	0.77	0.77	0.77
Mean (Median)	0.22 (0.22)	**0.27 (0.26)**	**0.19 (0.16)**	**0.33 (0.30)**
Heritability (S.E.)	0.25 (0.07)	**0.23 (0.05)**	0.09 (0.10)	**0.37 (0.10)**
*p*-value	0.00004	< 0.000002	0.17	0.0001

**Table 2:** Distribution and heritability of SBP slope for Replicate 19 of the simulated data

	**Complete Data**		**Missing Data**	
	
	**Method 1**	**Method 2**	**Method 1**	**Method 2**
*N*	1205	1516	1254	1548
Range	(-0.55, 1.60)	(-0.55, 2.23)	(-0.55, 1.60)	(-0.55, 2.31)
Std. Dev.	0.40	0.44	0.39	0.43
Mean (Median)	0.42 (0.40)	0.53 (0.53)	0.43 (0.42)	0.53 (0.53)
Heritability (S.E.)	0.60 (0.07)	0.74 (0.06)	0.57 (0.07)	0.70 (0.06)
*p*-value	< 0.0000001	< 0.0000001	< 0.0000001	< 0.0000001

**Table 3:** LOD scores from two-point linkage analysis for Framingham data (Method 2)

**Chromosome^A^**	**Marker**	**All Data **(*n *= 1579)	**Sex-Specific Data**	
			
			Female (*n *= 809)	Male (*n *= 770)
7 (70 cM)	GATA24D12	**2.24**	0.81	0.36
18 (107 cM)	ATA82B02	0.52	**2.25**	0.00

^A^Only chromosomes with LOD score > 2.0 are shown.

**Table 4:** Effect of MI on linkage results on chromosome 21 with different amounts of missing data

		**Max LOD Score**			
		
**%**^A^	**# of Missing Values per Subject**	**Two-Point**^B^		**Multipoint**	
		
		**Without MI**	**With MI**	**Without MI**	**With MI**
**0**	**0**	**9.66**^C^		**13.45 (52 cM)**^C^	
10	1	9.39	9.82	13.30 (52 cM)	13.32 (52 cM)
	2 or more	11.08	10.20	14.69 (51 cM)	13.51 (51 cM)
25	1	9.38	9.66	13.11 (52 cM)	13.30 (51 cM)
	2 or more	7.41	8.63	9.16 (49 cM)	11.67 (50 cM)

^A^Percentage of subjects with missing data. ^B^Two-point LOD scores were found at the same location. ^C^Reference values.
